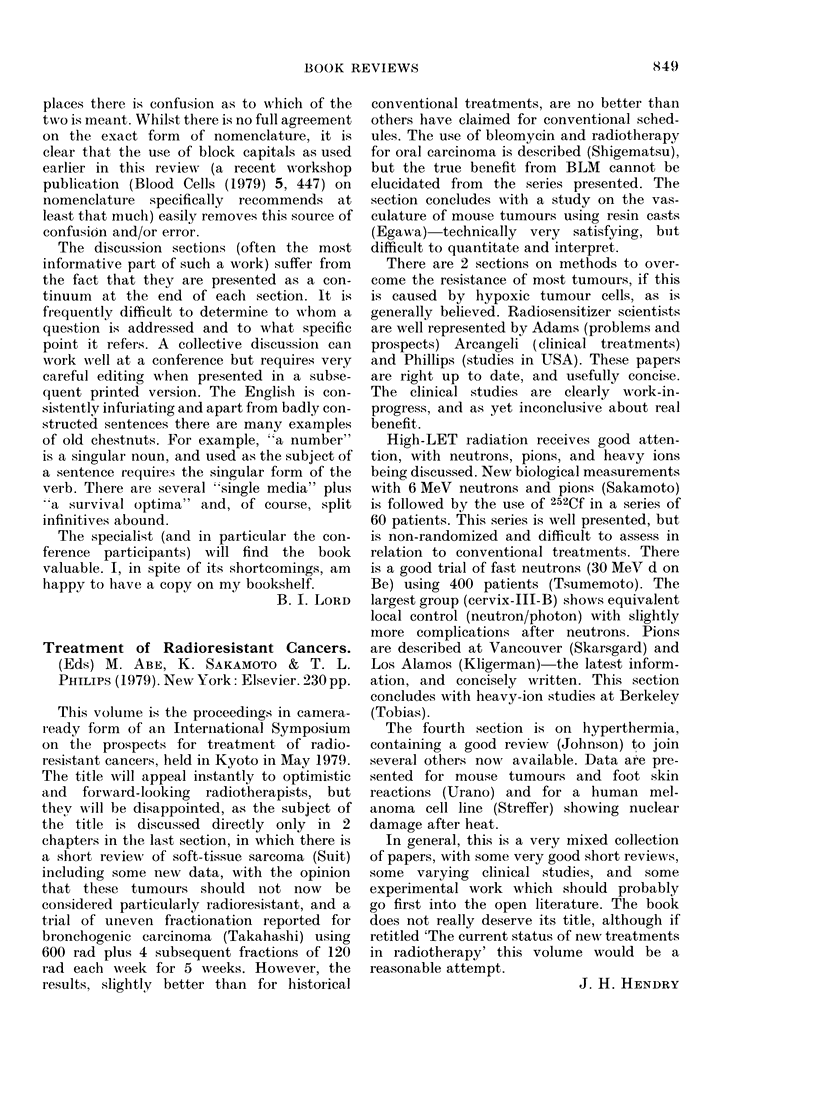# Treatment of Radioresistant Cancers

**Published:** 1980-05

**Authors:** J. H. Hendry


**Treatment of Radioresistant Cancers.** (Eds) M. ABE, K. SAKAMOTO & T. L. PHILIPS (1979). New York: Elsevier. 230 pp.

This volume is the proceedings in camera-ready form of an International Symposium on the prospects for treatment of radioresistant cancers, held in Kyoto in May 1979. The title will appeal instantly to optimistic and forward-looking radiotherapists, but they will be disappointed, as the subject of the title is discussed directly only in 2 chapters in the last section, in which there is a short review of soft-tissue sarcoma (Suit) including some new data, with the opinion that these tumours should not now be considered particularly radioresistant, and a trial of uneven fractionation reported for bronchogenic carcinoma (Takahashi) using 600 rad plus 4 subsequent fractions of 120 rad each week for 5 weeks. However, the results, slightly better than for historical conventional treatments, are no better than others have claimed for conventional schedules. The use of bleomycin and radiotherapy for oral carcinoma is described (Shigematsu), but the true benefit from BLM cannot be elucidated from the series presented. The section concludes with a study on the vasculature of mouse tumours using resin casts (Egawa)—technically very satisfying, but difficult to quantitate and interpret.

There are 2 sections on methods to overcome the resistance of most tumours, if this is caused by hypoxic tumour cells, as is generally believed. Radiosensitizer scientists are well represented by Adams (problems and prospects) Arcangeli (clinical treatments) and Phillips (studies in USA). These papers are right up to date, and usefully concise. The clinical studies are clearly work-in-progress, and as yet inconclusive about real benefit.

High-LET radiation receives good attention, with neutrons, pions, and heavy ions being discussed. New biological measurements with 6 MeV neutrons and pions (Sakamoto) is followed by the use of $^{252}$Cf in a series of 60 patients. This series is well presented, but is non-randomized and difficult to assess in relation to conventional treatments. There is a good trial of fast neutrons (30 MeV d on Be) using 400 patients (Tsumemoto). The largest group (cervix-III-B) shows equivalent local control (neutron/photon) with slightly more complications after neutrons. Pions are described at Vancouver (Skarsgard) and Los Alamos (Kligerman)—the latest information, and concisely written. This section concludes with heavy-ion studies at Berkeley (Tobias).

The fourth section is on hyperthermia, containing a good review (Johnson) to join several others now available. Data are presented for mouse tumours and foot skin reactions (Urano) and for a human melanoma cell line (Streffer) showing nuclear damage after heat.

In general, this is a very mixed collection of papers, with some very good short reviews, some varying clinical studies, and some experimental work which should probably go first into the open literature. The book does not really deserve its title, although if retitled 'The current status of new treatments in radiotherapy' this volume would be a reasonable attempt.

J. H. HENDRY